# Lack of right ventricular remodelling after surgical ventricular restoration

**DOI:** 10.1186/1532-429X-18-S1-P90

**Published:** 2016-01-27

**Authors:** Antonia Camporeale, Serenella Castelvecchio, Francesco Secchi, Paola Maria Cannao, Camilla Calvieri, Lorenzo Menicanti, Massimo Lombardi

**Affiliations:** Multimodality cardiac imaging section, San Donato Hospital, San Donato Milanese, Italy

## Background

Right ventricular (RV) dysfunction is an important predictor of long-term outcome in heart failure patients undergoing surgical ventricular restoration (SVR). RV assessment is therefore a pivotal step in the selection of patient candidates for SVR and during follow up. While the impact of SVR on left ventricle (LV) is currently described in terms of both functional and morphological parameters, RV changes are usually considered only from a functional point of view because of the complex chamber shape.

We aim to combine a morphological parameter, RV remodeling index (RV mass/volume ratio), with functional evaluation in order to describe and understand RV adaptation after SVR and the resulting impact on clinical outcome.

## Methods

A total of 32 patients (26 male, 6 female, mean age 65 ± 9) with previous myocardial infarction underwent Cardiac Magnetic Resonance (CMR) (1.5T) before and 6 months after SVR. RV and LV morpho-functional evaluations were performed by CMR. Doppler estimation of systolic pulmonary artery pressure (PAPs) was considered. Changes in NT-proBNP values, dosed before and 6 months after surgery at the time of scanning, were considered as markers of outcome.

## Results

Statistical significance of parameters changes after surgery was evaluated using Paired T-test or Wilcoxon Matched Pairs Signed-Ranks test as appropriate. Six months after SVR there was a significant reduction in NT-proBNP values compared with pre-surgical evaluation (p 0,02). LV EDV and LV ESV were significantly reduced (p < 0,01), while LV ejection fraction was significantly increased (p < 0,01). LV stroke volume did not show significant changes (p = 0,18). In the face of these results there was no significant variation in RV remodeling index (p = 0,35). RV mass remained unchanged (p = 0,48) despite a slight increase in RV EDV (p = 0,05), probably due to the loss of the compressive effect exerted by the dilated/aneurismal LV. Mean pre-surgical RV EF was 57% ± 11 and did not show significant variation after SVR (p = 0,54). Echocardiographic estimation of PAPs remained unchanged (p = 0,18). Correlations between variables were studied using Spearman Rank Correlation test. Changes in NT-proBNP levels were positively correlated with variations of PAPs. There was no correlation between changes in NT-proBNP levels and changes in RV ejection fraction (rho=-1,013, p = 0,49), LV ejection fraction (rho=-0,24, p = 0,49), RV stroke volume (rho=-0,24, p = 0,19) and LV stroke volume (rho=-0,21, p = 0,25).

## Conclusions

In patients with preserved RV systolic function undergoing SVR, there are no significant changes in RV structure and function after surgery. RV seems to behave as a ‘bystander’ in front of clinical improvement and LV modifications. Selection of patients with a ‘healthy’ RV is crucial for clinical success of SVR.

**Table 1:** CMR RV and LV morpho-functional parameters, Doppler estimation of PAPs and NT-proBNP values before and 6 months after SVR

	Before SVR (n = 32)	After SVR (n = 32)	P value
CMR LV EDV (ml)	213 ± 70	173 ± 55	<0,01
CMR LV ESV (ml)	157 ± 65	112 ± 43	<0.01
CMR LV EF (%)	27 ± 8	36 ± 10	<0.01
CMR LV SV (ml)	55 ± 21	61 ± 21	0.18
CMR LV MASS (g)	179 ± 37	160 ± 33	0.04
CMR RV EDV (ml)	88 ± 31	97 ± 31	0.05
CMR RV ESV (ml)	37 ± 16	42 ± 18	0.3
CMR RV EF (%)	57 ± 11	58 ± 10	0.54
CMR RV SV (ml)	51 ± 21	57 ± 20	0.3
CMR RV MASS (g)	54 ± 20	57 ± 15	0.48
CMR RV Rem.Ind.	0.92	0.97	0.35
ECHO PAPs (mmHg)	43 ± 15	40 ± 14	0.18
NT-proBNP (ng/ml)	2493	1460	0.02

**Figure 1:**
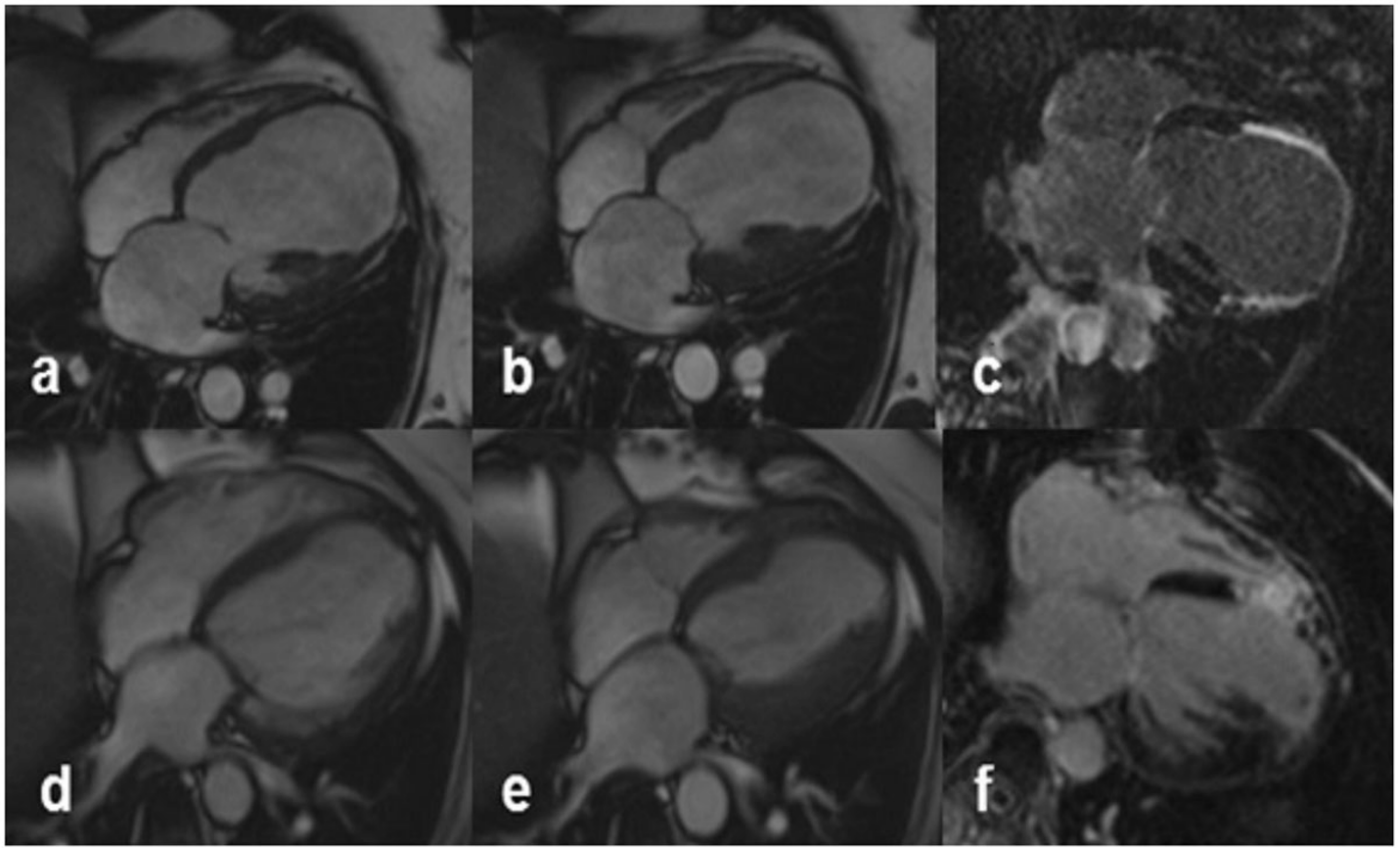
Figure 1

